# N-terminal pro atrial natriuretic peptide as a prognostic marker of cardiac resynchronization therapy recipients

**DOI:** 10.1016/j.ijcha.2023.101282

**Published:** 2023-10-24

**Authors:** Nobuhiko Ueda, Naoya Kataoka, Yuichiro Miyazaki, Keiko Shimamoto, Akinori Wakamiya, Kenzaburo Nakajima, Tsukasa Kamakura, Mitsuru Wada, Kohei Ishibashi, Kenichiro Yamagata, Yuko Inoue, Koji Miyamoto, Satoshi Nagase, Takeshi Aiba, Koichiro Kinugawa, Naoto Minamino, Kengo Kusano

**Affiliations:** a Department of Cardiovascular Medicine, National Cerebral and Cardiovascular Center, Suita, Japan; b Second Department of Internal Medicine, University of Toyama, Toyama, Japan; c National Cerebral and Cardiovascular Center Research Institute, Suita, Japan

## Abstract

**Background:**

Although the dynamic changes of atrial natriuretic peptide (ANP) expressions in a failing heart are well-documented, the clinical implications of detailed measurements of each ANP molecular form processed from proANP remain unclear.

**Methods:**

Patients screening was conducted on patients who were eligible for cardiac resynchronization therapy (CRT) between 2014 and 2019 in our institution. Blood samples and echocardiographic parameters were collected on the day before and six months after implantation. Total ANP, proANP, and N-terminal fragment of proANP (NT-proANP) were examined as predictive biomarkers for cardiac death, left ventricular assist device implantation, and heart failure hospitalization following CRT implantation.

**Results:**

A total of 86 subjects (mean age 70 years, 64 males) who underwent successful CRT implantation were enrolled. Plasma levels of total ANP, proANP, and NT-proANP were not normally distributed [25.8 pM (interquartile range: 11.1–53.1), 2.2 pM (1.0–5.4), and 4.1 nM (2.4–7.1), respectively]. Over a median follow-up of 2.7 years, 31 patients (2 deaths and 29 heart failure hospitalizations) reached the endpoints. Among the different ANP forms, only NT-proANP emerged as an independent predictor of the composite outcome (adjusted odds ratio of 2.542 in those with levels above vs. below the median, 95 % confidence interval 1.151–5.615, *p* = 0.021). NT-proANP levels were associated with left atrial volume and left diastolic functional parameters and decreased in response to echocardiographic improvements at six months post-implantation (16 ± 44 % decrease in responders vs 18 ± 60 % increase in non-responders, *p* = 0.005).

**Conclusion:**

Pre-implantation NT-proANP levels could serve as a predictive factor for clinical outcomes in recipients of CRT.

## 1 Introduction

Cardiac resynchronization therapy (CRT) is a well-established therapeutic strategy for individuals suffering from advanced heart failure. Nevertheless, robust biomarkers capable of accurately predicting clinical outcomes remains unidentified. In our recent study, we introduced a novel parameter known as the cyclic guanosine monophosphate (cGMP) to mature brain natriuretic peptide (BNP) ratio, which provides a potential prognostic indicator in CRT recipients [1]. The utility of similar index was substantiated through its evaluation within the context of the PARADIGM-HF trial [2]. The cGMP to mature BNP ratio is considered to mirror the physiological activity of natriuretic peptides (NPs); therefore, elevated ratios may serve as predictive markers for favorable outcomes. However, it is crucial to acknowledge that NPs involved in cGMP production encompass not only BNP but also atrial NP (ANP). Consequently, conducting comprehensive investigations into the dynamics of plasma ANP fluctuations is imperative to gain insights into the prognostic implications of NPs in CRT recipients.

In humans, four endogenous molecular forms of ANP have been identified: precursor ANP (proANP), mature ANP which includes α-ANP and β-ANP as biologically active forms, and N-terminal fragment (NT) of proANP (NT-proANP) which is an inactive form [3]. ProANP which is the majority of ANP stored in the atrial granules is converted to NT-proANP and mature ANP when secretion in healthy subjects, whereas α-ANP and β-ANP are produced in the granules in patients with heart failure [4], [5]. Individual measurements of these ANP forms have not been performed by lack of assay methods specific to each ANP form, and the alterations of these ANP forms related to heart failure remain unclear. Therefore, we recently developed a proANP-specific chemiluminescent enzyme immunoassay (CLEIA) and total ANP CLEIA [6]. Additionally, we developed a sensitive radioimmunoassay (RIA) for NT-proANP in this study.

In the present study, we aimed to clarify the correlations between plasma levels of individual ANP forms and clinical outcomes subsequent to CRT implantation.

## 2 Methods

### 2.1 Study population

The study was conducted at our institute between 2014 and 2019, with consecutive candidates for CRT enrolled prospectively. To ensure the inclusion of appropriate subjects, the following criteria were applied: 1) New York Heart Association functional class II-IV, 2) left ventricular ejection fraction ≤ 35 %, and 3) a QRS duration ≥ 120 msec [7]. To maintain the study's integrity and validity, certain exclusions were implemented. Patients with the following conditions were excluded: 1) decompensated heart failure, 2) invasive cardiovascular therapies within three months before the enrolment, 3) severe aortic stenosis, 4) hemodialysis, and 5) administration of angiotensin receptor neprilysin inhibitors. This study conducted in accordance with the Declaration of Helsinki was approved by the Institutional Research Board of the National Cerebral and Cardiovascular Center, Suita, Japan (M25-052), and written consent was obtained from all patients (UMIN R000038927).

### 2.2 Measurements of plasma levels of three ANP forms

Plasma levels of total ANP, proANP, and NT-proANP were evaluated as predictive markers. Plasma samples were collected in plastic tubes containing ethylenediaminetetraacetic acid disodium salt (1.25 mg/ml) and aprotinin (500kallikrein inhibitor units/ml) (NP-EA0305, Nipro, Osaka, Japan) the day before CRT implantation and 6 months following the implantation, which were aliquoted and stored at −80 °C before use. Total ANP and proANP were measured using our recently developed plate-based [6] CLEIAs employing recombinant proANP as a standard. In the total ANP CLEIA, α-ANP, β-ANP, and proANP were recognized at a molar ratio of 1:2:1. NT-proANP was measured by the newly developed radioimmunoassay (RIA) method, which were described in the supplementary information.

### 2.3 Clinical characteristics

The following clinical data were extracted from the patients‘ medical records: age, gender, underlying heart disease, comorbidities, medication, laboratory data including total BNP and NT-proBNP, echocardiographic parameters, and 12-lead electrocardiogram.

### 2.4 CRT implantation procedure

CRT devices were implanted using the standard transvenous approach. The procedure involved the placement of a right ventricular (RV) lead was positioned at the RV septal apex. Subsequently, a left ventricular lead was implanted in a suitable branch of the coronary venous system, specifically in the lateral, posterolateral, or anterolateral region. The selection of the implantation site was based on the ability to achieve an acceptable pacing threshold while avoiding diaphragmatic pacing.

### 2.5 CRT optimization

CRT devices were programmed to DDD(R) for sinus rhythm and VVI(R) for atrial fibrillation. To optimize the atrioventricular delay, echocardiography guidance was employed. This involved assessing various parameters to determine the optimal atrioventricular delay that would provide the maximum left ventricular filling time. The goal was to avoid interference from atrial wave activity and diastolic mitral regurgitation. Additionally, the inter-ventricular delay was adjusted based on the pulsed wave left ventricular outflow tract velocity.

### 2.6 Clinical endpoints

The evaluation of echocardiographic response was conducted at the 6-month mark following implantation. A patient was classified as a CRT responder if there was a reduction of left ventricular end-systolic volume ≥ 15 % and/or an improvement in left ventricular ejection fraction > 10 % compared to the pre-implantation values. The primary clinical outcome was defined as a composite endpoint, comprising cardiac death, left ventricular assist device implantation, or unplanned heart failure hospitalization. The secondary endpoints were defined as individual events within the composite outcome.

In addition to echocardiographic and clinical parameters, biomarkers with predictive potential were also assessed. The evaluation focused on analyzing the association between the time-course changes in these biomarkers following implantation and the corresponding echocardiographic responses.

Each endpoint was separately analyzed in terms of bundle branch block or QRS width types [8] to assess the significance of NT-proANP levels within each condition.

### 2.7 Statistical analysis

The results were presented as the mean ± standard deviation or as the median and interquartile range (IQR) for continuous data, depending on the nature of the continuous data. Categorical data were expressed as counts and percentages. Statistical comparisons between categorical variables were performed using the χ2 test or Fisher’s exact test when appropriate. For continuous variables, the Wilcoxon rank-sum test was utilized for group comparisons. Survival analysis was conducted using the Kaplan-Meier method, with the comparison divided by the median of each biomarker. The log-rank test was employed to assess the differences in survival curves between the groups. The effects of covariates on the time to endpoint were investigated using a Cox proportional hazards model. Additionally, a multiple linear regression model was employed to test the influence of multiple covariates on the study outcomes. A value of *p* < 0.05 was taken as a threshold for statistical significance. All analyses were performed using JMP 14 software (SAS Institute Inc., Cary, NC, USA).

## 3 Results

### 3.1 Baseline clinical characteristics

**Fig. 1:**
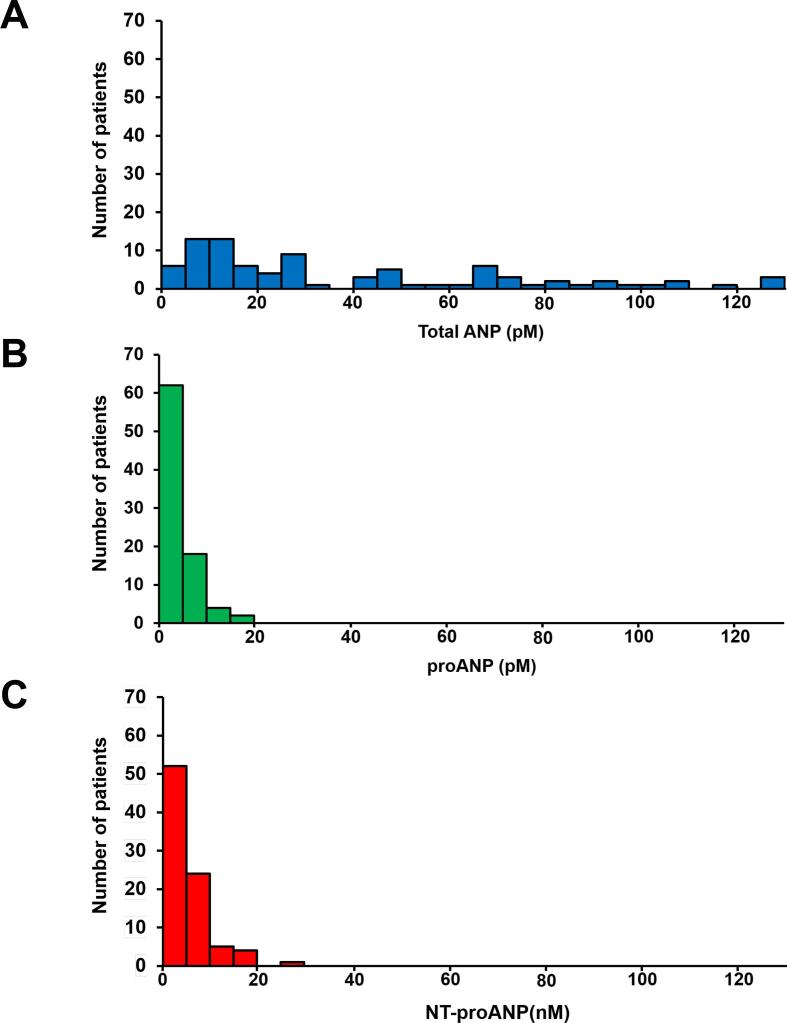
**The patient distribution in each plasma level of ANP molecular form.** A. Total ANP: the median of 25.8 pM [IQR: 11.1 – 53.1 pM] B. ProANP: the median of 2.2 pM [IQR: 1.0 – 5.4 pM] C. NT-proANP: the median of 4.1 nM [IQR: 2.4 – 7.1 nM] ANP, atrial natriuretic peptide; IQR, interquartile range; NT-proANP, N-terminal proANP.

Total of 86 patients (64 male) were prospectively enrolled in the present study, as detailed in Table 1. The mean age of the participants was 70 [57 – 76] years. Among the enrolled patients, 17 patients (20 %) had ischemic etiology, and 67 % of the subjects exhibited a left bundle branch block. Non-normal patient number distributions observed for the plasma total ANP, proANP, and NT-proANP levels prior to CRT implantation in Fig. 1.

**Table 1:** Clinical characteristics.

Variable	Overall (N = 86)
Age (years)	70 [57 – 76]
Male, n (%)	64 (74)
BMI (kg/m^2^)	21.7 ± 3.2
CRT-D, n (%) / CRT-P, n (%)	68 (71) / 18 (21)
*Comorbidities*	
Ischemic etiology, n (%)	17 (20)
Diabetes mellitus, n (%)	27 (31)
Chronic kidney disease, n (%)	41 (49)
*NYHA functional class*	
II, n (%)	33 (38)
III, n (%)	47 (55)
IV, n (%)	6 (7)
*Medications*	
ACEi or ARB, n (%)	61 (71)
β-blockers, n (%)	75 (87)
Diuretics, n (%)	73 (85)
Amiodarone, n (%)	30 (35)
Sotalol, n (%)	5 (6)
*Echocardiographic parameters*	
LV ejection fraction (%)	26 [20 – 34]
LV end diastolic volume (ml)	173 [129 – 239]
LV end systolic volume (ml)	123 [90 – 202]
Left atrial volume (ml)	108 [62 – 146]
Mitral valve regurgitation (grade)	1.5 [1], [2], [3]
Tricuspid valve regurgitation (grade)	1 [1], [2]
E/A	0.9 [0.6–2.2]
E/e’	13.0 [9.1–17.3]
*Electrocardiographic parameters*	
Persistent atrial fibrillation, n (%)	20 (23)
LBBB, n (%)	58 (67)
QRS duration (msec)	156 ± 29
QRS axis (degree)	0 [-37 – 25]
PR interval (msec)	196 [180 – 227]
Left axis deviation, n (%)	24 (28)
*Laboratory data*	
Total bilirubin (mg/dl)	0.7 [0.5–1.0]
Hemoglobin (g/dl)	12.8 ± 1.7
Estimated GFR (ml/min/1.73 m^2^)	51.1 ± 19.7
Sodium (mEq/L)	140 [138 – 142]

ACE, angiotensin converting enzyme inhibitor; ARB, angiotensin receptor blocker; BMI, body mass index; CRT-D, cardiac resynchronization therapy defibrillator; CRT-P, cardiac resynchronization therapy pacemaker; GFR, glomerular filtration rate; LBBB, left bundle branch block; LV, left ventricular; NYHA, New York Heart Association.

### 3.2 Impacts of each natriuretic peptide level for primary outcome

**Fig. 2:**
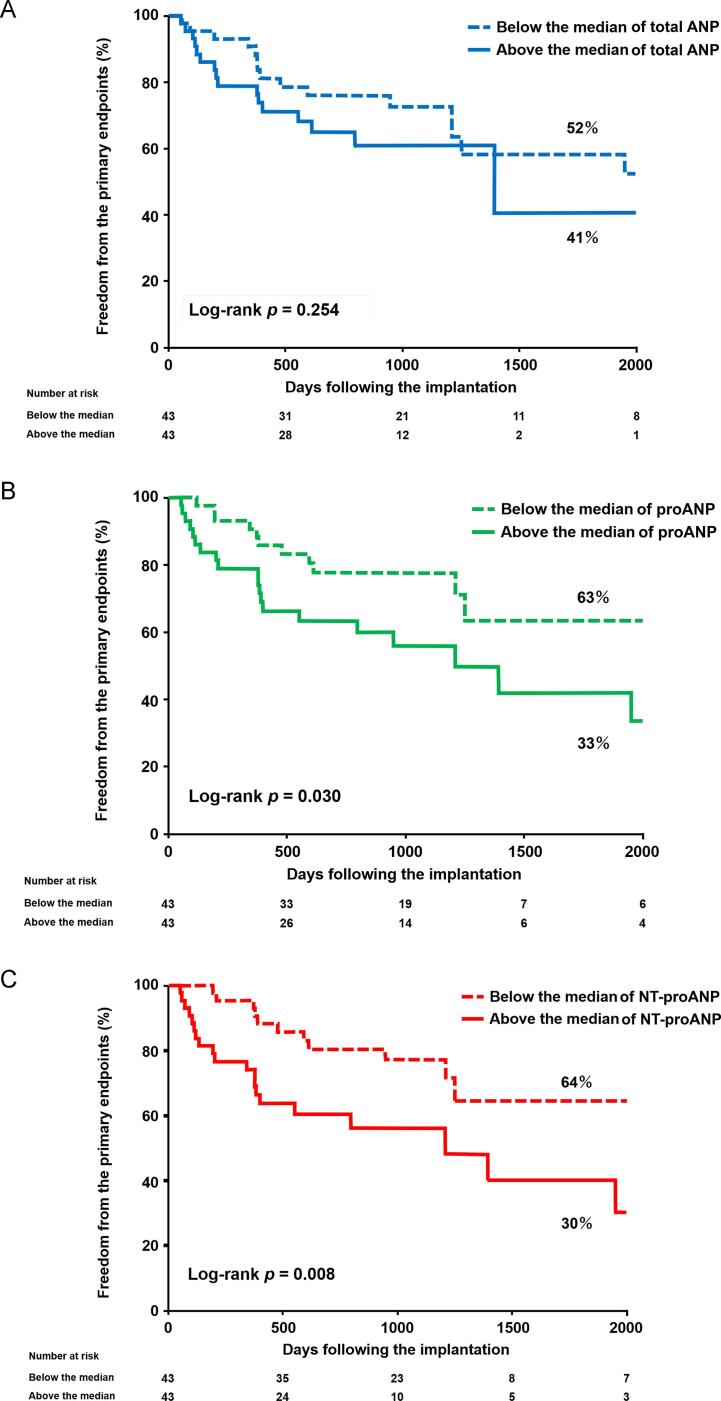
**Kaplan-Meier analyses of freedom from the primary outcome stratified by the median of total ANP (A), proANP (B), and NT-proANP (C).** Dotted lines indicate below the median groups; solid lines, above the median groups. Abbreviations are same as Fig. 1.

**Fig. 3:**
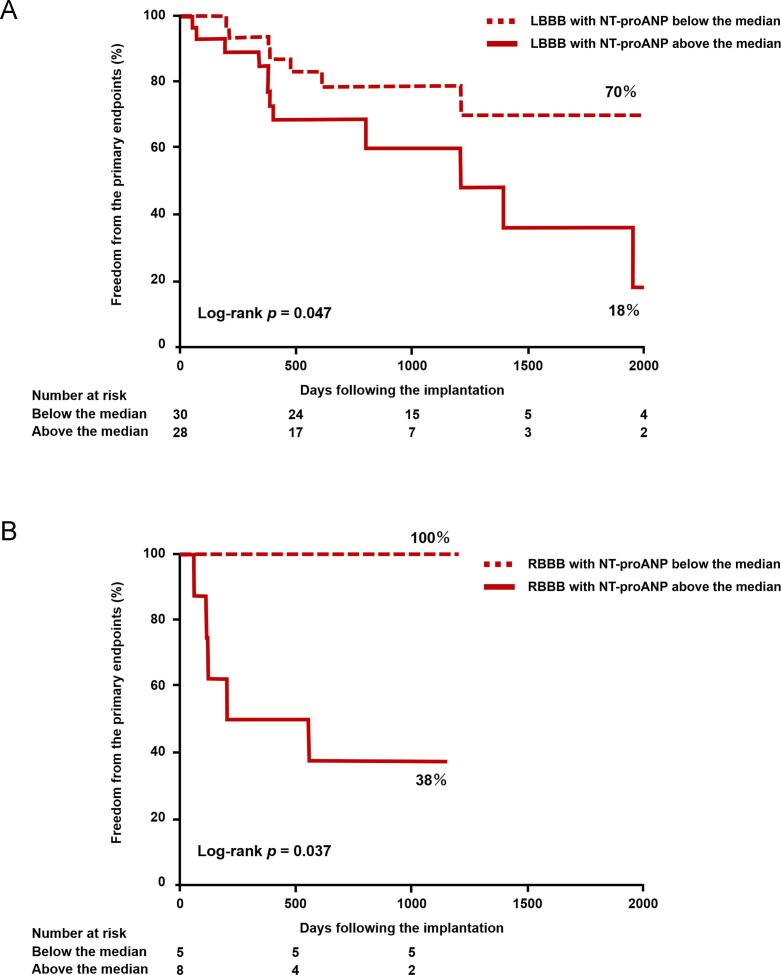

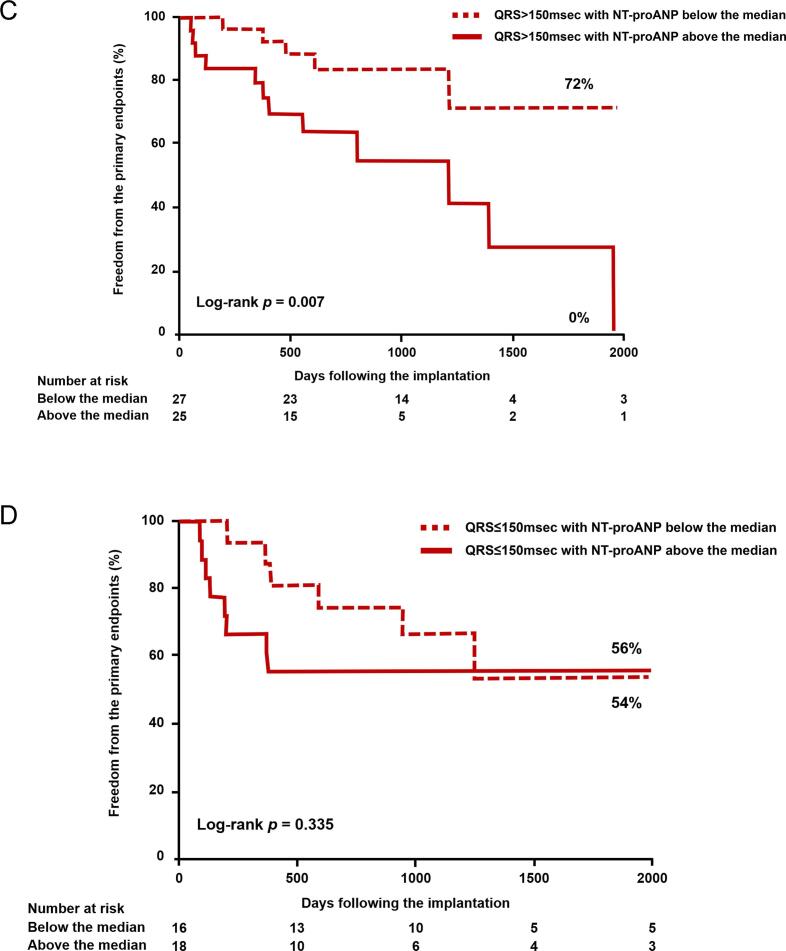
**Stratified analysis of Kaplan-Meier curves for freedom from the primary outcome by NT-proANP median, according to bundle branch block type or QRS Width.** Left bundle branch block, Right bundle branch block, QRS width > 150 milli second. A. QRS width ≤ 150 milli second. Dotted lines indicate below the median groups; solid lines, above the median groups. LBBB; left bundle branch block, RBBB; right bundle branch block.

During a median follow-up of 794 days after CRT implantation (IQR: 384 – 1191 days), the primary outcome was observed in 31 subjects (2 deaths and 20 heart failure hospitalizations). Kaplan-Meier curves representing the primary outcome, stratified by the median level of each ANP form before CRT implantation, were presented in Fig. 2. While the total ANP level (Fig. 2-A) did not show discriminatory ability for the primary outcome using the median cutoff, levels below the median of proANP (Fig. 2-B) and NT-proANP (Fig. 2-C) levels were associated with favorable outcomes. Among the patients with left bundle branch block, right bundle branch block, QRS duration > 150 ms, those with NT-proANP levels above the median exhibited a higher risk of the primary endpoint compared to patients with levels below the median. However, there was no significant difference observed among patients with a QRS duration ≤ 150 ms (Fig. 3). Patients with NT-proANP levels below the median demonstrated similar outcomes compared to patients with NT-proANP levels above the median in terms of cardiac death or left ventricular assist device implantation (87 % vs. 56 %, Log-rank *p* = 0.379). However, the former group experienced significantly more favorable outcomes in terms of hospitalization due to worsening heart failure (68 % vs. 30 %, Log-rank *p* = 0.002).

Table 2 presented the results of the Cox proportional hazards analysis for the primary outcome. Univariable analysis showed that body mass index > 22 kg/m^2^, diabetes mellitus, hemoglobin, and plasma levels above the median of proANP and NT-proANP before CRT implantation were associated with the primary endpoint. In the analysis adjusted for body mass index, diabetes mellitus, and hemoglobin, NT-proANP emerged as a predictor of the primary outcome, whereas proANP did not. Interestingly, total BNP or NT-proBNP were not associated with the primary endpoint.

**Table 2:** Univariate and multivariate analysis to predict cardiac death or heart failure hospitalization.

	Univariate analysis			Natriuretic peptides adjusted for BMI > 22, diabetes mellitus, and hemoglobin		
	Hazard Ratio	95 %CI	*p* value	Hazard Ratio	95 %CI	*p* value
Age (1 yr increase)	1.004	0.977–––1.034	0.792			
Male	0.563	0.270–––1.176	0.126			
BMI > 22 kg/m^2^	0.306	0.136–––0.689	0.004			
*Comorbidity*						
Ischemic etiology	1.597	0.713–––3.575	0.255			
Diabetes mellitus	2.497	1.232–––5.064	0.011			
Chronic kidney disease	1.888	0.894–––3.989	0.096			
*Medications*						
ACEi or ARB	0.774	0.364–––1.645	0.505			
Beta blocker	0.795	0.304–––2.080	0.640			
Diuretics	1.087	0.377–––3.137	0.877			
*Echocardiographic parameters*						
Left atrial volume (1 ml increase)	1.002	0.996–––1.008	0.400			
LV end diastolic volume (1 ml increase)	0.999	0.996–––1.003	0.737			
LV end systolic volume (1 ml increase)	0.999	0.995–––1.003	0.756			
LV ejection fraction (1 % increase)	0.988	0.949–––1.026	0.551			
*Electrocardiographic parameters*						
Persistent atrial fibrillation	0.783	0.320–––1.917	0.592			
QRS duration (1 ms increase)	0.995	0.984–––1.007	0.449			
Non-LBBB	1.328	0.643–––2.742	0.443			
*Laboratory data*						
Total bilirubin (1 mg/dl)	0.806	0.339–––1.632	0.579			
Hemoglobin (1 g/dl increase)	0.797	0.643–––0.990	0.040			
eGFR (1 ml/min/1.73 m^2^ increase)	0.991	0.973–––1.010	0.357			
Sodium (1 mEq/L increase)	0.949	0.876–––1.041	0.253			
Potassium (1 mEq/L increase)	0.917	0.405–––2.026	0.833			
*Natriuretic peptides above each median*						
total BNP	2.145	1.038–––4.429	0.039	1.749	0.827–––3.695	0.143
proBNP	2.344	1.120–––4.905	0.024	1.933	0.901–––4.147	0.091
NT-proBNP	1.573	0.770–––3.211	0.214			
total ANP	1.518	0.737–––3.128	0.257			
proANP	2.215	1.061–––4.627	0.034	2.027	0.931–––4.415	0.075
NT-proANP	2.621	1.250–––5.496	0.011	2.542	1.151–––5.615	0.021

CI = Confidence Interval. Other abbreviations are same as Table 1.

### 3.3 Correlation between NT-proANP and clinical parameters

The relationships of NT-proANP levels with clinical characteristics, as well as echocardiographic and electrocardiographic parameters, were assessed (Table 3). Importantly, significant correlations were observed between NT-proANP levels and left ventricular ejection fraction, left atrial volume, mitral regurgitation grade, E/A ratio, and E/e’ ratio. However, no significant associations were found with electrocardiographic parameters.

**Table 3:** Relationships between NT-proANP and clinical characteristics.

	r	*p* value
Age (years)	0.178	0.101
BMI (kg/m^2^)	0.161	0.138
*Echocardiographic parameters*		
LV ejection fraction (%)	0.228	0.035
LV end diastolic volume (ml)	0.137	0.208
LV end systolic volume (ml)	0.157	0.150
Left atrial volume (ml)	0.264	0.015
Mitral regurgitation (grade)	0.327	0.002
E/A	0.300	0.025
E/e’	0.290	0.010
*Electrocardiographic parameters*		
QRS duration (ms)	0.010	0.361
QRS axis (degree)	0.109	0.319
PR interval (ms)	0.144	0.350

Abbreviations are same as Table 1.

### 3.4 NT-proANP dynamics and echocardiographic response

**Fig. 4:**
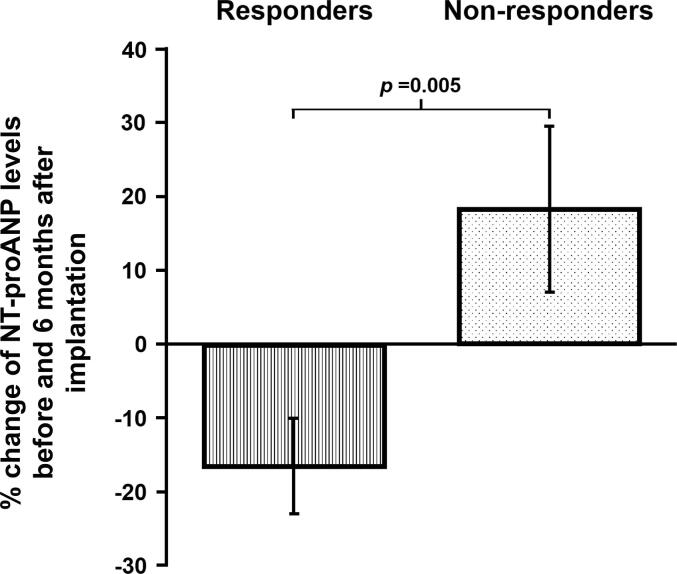
**Percent change of NT-proANP levels before and 6 months after implantation in CRT responders and non-responders,** NT-proANP decreased significantly in CRT responders six months following implantation compared with those of non-responders. The striped bar indicates the NT-proANP change rate compared those before with those after implantation in responders; the dotted bar, those in non-responders. Values are means ± standard error.

A total of 47 patients met the criteria for CRT responder. Above-median NT-proANP levels demonstrated a tendency toward a positive correlation with improvements in LV ejection fraction and/or LV end-systolic volume (*p* = 0.052). NT-proANP levels before CRT implantation did not differ significantly between responders and non-responders (3.03 [1.76 – 5.71] nM vs. 4.91 [2.66 – 7.67] nM. *p* = 0.097). However, CRT responders exhibited a decrease in NT-proANP levels 6 months after implantation, while non-responders showed the opposite pattern of changes (16 ± 44 % decreased vs 18 ± 60 % increased, *p* = 0.005) (Fig. 4).

## 4 Discussion

### 4.1 Main findings

The present study revealed novel findings regarding the relationship between plasma levels of ANP forms and a failing heart in patients undergoing CRT implantation, as followed; 1) Low plasma levels of NT-proANP before CRT implantation predicted favorable outcomes after the implantation, 2) NT-proANP levels were positively correlated with left atrial volume and left ventricular parameters associated with diastolic dysfunction, and 3) plasma levels of NT-proANP decreased in conjunction with echocardiographic responses to CRT at six months after implantation.

### 4.2 Biosynthesis and molecular forms of ANP

In humans, ANP exists in four distinct endogenous molecular forms; proANP, mature ANP, and NT-proANP, with β-ANP being a minor form [6]. Among these forms, proANP is the primary storage form of ANP in atrial granules, and undergoes conversion to mature ANP and NT-proANP through the action of corin during secretion in the normal atrium. [4], [5] Notably, the large secretory granules containing ANP are found in atrial cardiomyocytes but not in ventricular cardiomyocytes [9]. The observation suggests that the regulation of ANP storage, conversion, and secretion differs between the atrium and ventricle. In the failing heart, on the other hand, corin is internalized and α-ANP and β-ANP are stored in the granules in the atrium, while ANP biosynthesis is augmented and uncleaved proANP is secreted from the ventricle. Although the detailed mechanism of ANP biosynthesis and secretion in the atrium and ventricle has yet to be elucidated, these facts suggest that concentrations of these molecular forms of ANP in the circulation reflects pathophysiological status of cardiomyocytes in patients with heart failure.

### 4.3 Differences in the prognostic value among each ANP molecular form

Previous studies have demonstrated that NT-proANP can serve as a diagnostic biomarker and a predictor of heart failure [10], [11], [12]. Additionally, NT-proANP may have superior prognostic power compared to NT-proBNP, particularly in obese patients [13]. To the best of our knowledge, this paper represented the first report investigating the clinical implications of NT-proANP in CRT recipients.

In a recent study by Michel T. et al., it was shown that corin cleaves proANP to generate NT-proANP and mature ANP. which are subsequently degraded by meprin B and neprilysin, respectively [14]. One hypothesis to explain the differences in predictive abilities is the variation in the proteolytic enzymes responsible for generating and degrading each ANP form. In heart failure with systolic dysfunction, both corin and meprin B activities are suppressed in the atrium compared to healthy subjects [14]. Consequently, NT-proANP, which reflects the activities of these two enzymes as the upper and lower parts of the proteolytic cascade for ANPs, might predict clinical outcomes more accurately than proANP, which only reflect corin activity as the upper part. Total ANP represent the sum of proANP, α-ANP and β-ANP levels since it is measured using antibodies targeting the C-terminal tail and the ring portion of ANP, which may result in an ambiguous index because of the summation of more generating and degrading pathways. Therefore, the detailed measurements of each ANP form in this study can provide more accurate predictive indices compared to total ANP. Another possible explanation lies in the difference in plasma levels among the three ANP forms. The approximately one-thousand-fold lower plasma levels of proANP compared to NT-proANP may impact the statistical power.

### 4.4 Clinical impacts of NT-proANP measurement before and after CRT implantation

In our study, NT-proANP showed associations with left atrial volume, severity of mitral regurgitation, E/A ratio, and E/e’ ration, which are indicators of left atrial function and left ventricular diastolic function. Left ventricular diastolic dysfunction leads to an elevation in left atrial pressure [15]. Therefore, plasma levels of NT-proANP increase in line with left atrial overload, which is further supported by the specific expression of ANP messenger RNA in the atrium [2]. Mitral regurgitation and left atrium enlargement, both of which contribute to left atrial overload, have been identified as the risk factors for death or unplanned cardiovascular hospitalization in CRT recipients [15], [16]. Hence, our findings demonstrating that high levels of NT-proANP before CRT implantation predicted unfavorable outcomes were consistent with these previous studies. Notably, individuals with right bundle branch block whose NT-proANP levels remained below the median experienced complete freedom from adverse events throughout the follow-up period. These findings suggest the potential utility of stratifying candidates for CRT who present with right bundle branch block.

In the follow-up data, we observed a decrease in NT-proANP levels in patients who responded to CRT therapy, whereas non-responders exhibited an increase. These findings suggest that NT-proANP expression fluctuates alongside the reverse remodeling of both the left atrium and left ventricle following CRT implantation. Monitoring NT-proANP levels post-implantation may serve as a viable alternative to frequent echocardiographic assessments.

### 4.5 The implication of both ANP and BNP measurements

Mature BNP and N-terminal proBNP measurements are widely used in heart failure management. In a previous study, we demonstrated the significance of measuring each BNP molecular form in CRT recipients [1]. Importantly, plasma levels of proBNP, mature BNP, and NT-proBNP alone did not predict clinical outcomes or echocardiographic responses. However, combining mature BNP measurement with cyclic guanosine monophosphate, the second messenger of natriuretic peptides, was found to predict heart failure rehospitalization [1]. On the other hand, ANP molecular forms, particularly NT-proANP, were able to independently predict cardiovascular death and worsening heart failure in our current study. Therefore, NT-proANP measurements may be more easily applicable in clinical practices. These results suggest that the regulation of ANP forms may play a more critical role in advanced failing heart requiring CRT implantation in terms of NPs biosynthesis.

## 5 Limitations

This study has several limitations. Firstly, the sample size of enrolled subjects was small, and the study was conducted at a single-center, limiting the generalizability of the findings. Secondly, the mechanisms of underlying the regulation of each ANP form processing in relation to atrial overload or ventricular diastolic dysfunction remained a topic of future investigation. Thirdly, the study population lacked individuals who did not undergo CRT implantation making it unclear whether the predictive power observed in this study is applicable to a broader population with advanced heart failure. Fourthly, β-ANP levels were not measured in this study due to lack of sufficient plasma sample for condensation and measurements. Finally, the administration of sacubitril/valsartan, which is a standard therapeutic strategy for systolic dysfunction, may affect the proteolytic process of ANP. However, no patients receiving this treatment were included in our study.

## 6 Conclusions

A high plasma level of NT-proANP before the procedure was associated with unfavorable outcomes following CRT implantation. Details measurements of each ANP molecular form could provide valuable insights into heart failure with systolic dysfunction.

**Funding**

This work was supported by the Intramural Research Fund of the National Cerebral and Cardiovascular Center [grant number 25-4-7, Kengo Kusano；22-1-4 and 27-1-5, Naoto Minamino].

## Declaration of Competing Interest

The authors declare that they have no known competing financial interests or personal relationships that could have appeared to influence the work reported in this paper.
