# Supplementary material for: N-terminal pro atrial natriuretic peptide as a prognostic marker of cardiac resynchronization therapy recipients
Source: Int J Cardiol Heart Vasc. 2023 Oct 24;49:101282. doi: 10.1016/j.ijcha.2023.101282 (PMC10613895; doi:10.1016/j.ijcha.2023.101282)
Supplement: Supplementary Data 1 [file mmc1.docx]

**Supplementary Methods**

**Measurements of NT-proANP.**

NT-proANP levels were measured by the competitive radioimmunoassay (RIA) using a newly developed polyclonal antibody (#593-10) against N-terminal sequence [1-31] of proANP (proANP[1-31]), which was prepared by immunizing rabbits with proANP(1-31)-keyhole limpet hemocyanin conjugate and used at a final dilution of 1:90,000. RIA for NT-proANP was performed as reported with some modifications.^1^ C-terminally tyrosinated proANP[1-31] (proANP[1-31]-Tyr) was radioiodinated by the lactoperoxidase methos, purified by reverse phase high performance liquid chromatography, and used as a tracer. FLAG tag-linked proANP and proANP[1-31]-Tyr and were used as standards. Plasma samples (20µL) or standards were mildly oxidized their methionine residues by the action of 250µL of 0.06% hydrogen peroxide in 1M formic acid at 20ºC for 2 hours, lyophilized, dissolved in 400µL of a RIA buffer, and submitted to the RIA. Half-maximum tracer binding was observed at 50 pM and detection limit was 3 pM. The NT-proANP RIA recognizes NT-proANP and proANP at an equimolar ratio.

**References**

1. Katafuchi T, Kikumoto K, Hamano K, Kangawa K, Matsuo H and Minamino N. Calcitonin receptor-stimulating peptide, a new member of the calcitonin gene-related peptide family. Its isolation from porcine brain, structure, tissue distribution, and biological activity. *J Biol Chem*. 2003;278:12046-54.
